# Beckwith Wiedemann imprinting defect found in leucocyte but not buccal DNA in a child born small for gestational age

**DOI:** 10.1186/1471-2350-13-99

**Published:** 2012-11-01

**Authors:** Rinki Murphy, Deborah Mackay, Ed A Mitchell

**Affiliations:** 1 Department of Medicine, FMHS, University of Auckland, Auckland, New Zealand; 2 Faculty of Medicine, University of Southampton, Southampton, UK; 3 Faculty of Medical and Health Sciences, University of Auckland, Private Bag 92019, Auckland, New Zealand

**Keywords:** Insulin like growth factor 2 gene, Small for gestational age, Beckwith Wiedemann syndrome

## Abstract

**Background:**

Loss of methylation (LOM) at imprinting control region (ICR) 1 or LOM at ICR 2 on chromosome 11p15 in leucocyte DNA is commonly used to diagnose the imprinting disorders Silver Russell syndrome (SRS) characterized by growth restriction or Beckwith Wiedemann syndrome (BWS) characterized by overgrowth, respectively.

**Case presentation:**

A child was normally conceived and born by caesarian section to a healthy 19 year old smoking mother (G2P1) at 38 weeks gestation, with SGA (birthweight SDS −2.44), placenta weight 250g (normal histology), with an umbilical hernia and transient neonatal hypoglycemia but no other features of BWS.

The methylation status at 11p15 region was initially investigated by multiplex ligation dependent probe amplification (MLPA). Subsequently, methylation-specific (ms) PCR was performed to screen for this and other imprinted loci abnormalities at PLAG1 (6q24), IGF2R (6q27), GRB10 (7p12), PEG1/MEST (7q32), DLK1 (14q32), SNRPN (15q11); PEG3 (19q32), NESPAS/GNAS (20q13).

Leucocyte DNA methylation was normal at ICR1 but markedly reduced at ICR2 using both MLPA and ms-PCR, and no other anomalies of imprinting were detected. Buccal DNA methylation was normal at all imprinted sites tested.

**Conclusion:**

This is the first report of an isolated LOM at ICR2 in leucocyte but not buccal DNA in a normally conceived singleton SGA child without overt SRS or BWS.

## Background

Two clusters of imprinted genes critical to early human growth are expressed in a parent of origin-specific manner under the control of imprinting control regions (ICR) on chromosome 11p15. These are CpG-rich regions that are differentially methylated on the maternally and paternally derived chromosomes (differentially methylated regions or DMR). ICR1 (or H19 DMR) is methylated on the paternally-inherited allele and regulates expression of insulin-like growth factor 2 (*IGF2*) and H19. ICR2 (or KvDMR) is methylated on the maternally-inherited allele and regulates expression of cell cycle regulator *CDKN1C*[1]. Loss of methylation (LOM) of ICR1 is a major cause of Silver Russell syndrome (SRS: OMIM 180860), characterized by intrauterine and postnatal growth restriction and a variable expression of limb asymmetry, relative macrocephaly, triangular face and feeding difficulties in early childhood [2,3]. LOM of ICR2 is a major cause of Beckwith Wiedemann syndrome (BWS: OMIM 130650), characterized by fetal overgrowth and a variable expression of other features such as macroglossia, abdominal wall defects and predisposition to embryonal tumours [1].

Cases of SRS and BWS with hypomethylation at both ICR1 and ICR2 have recently been described [4-6]. Furthermore, apparently healthy monozygotic twins of either SRS [2,4] or BWS [7] probands have been found to have similar methylation defects as their affected twins in leucocyte but not buccal DNA. Additional hypomethylation at multiple imprinted loci outside of 11p15 have been identified in leucocyte DNA from some patients with BWS or SRS [5-7]. LOM at ICR2 as part of multi-locus hypomethylation is also seen in leucocyte DNA from a proportion of cases with transient neonatal diabetes due to LOM of 6q24 [8,9]. Here we report a singleton female patient born small for gestational age (birthweight SDS −2.44) with isolated LOM at ICR2 in leucocyte but not buccal DNA who had transient neonatal hypoglycemia and an umbilical hernia, but no other features of BWS or SRS.

## Case presentation

We identified this case after screening leucocyte DNA from 366 phenotypically normal children, who took part in the ABC study [10], for methylation abnormalities in ICR1 and ICR2, as part of our investigation into the role of common epigenetic variation in determining risk of low birthweight (manuscript under review).

**Figure 1:**
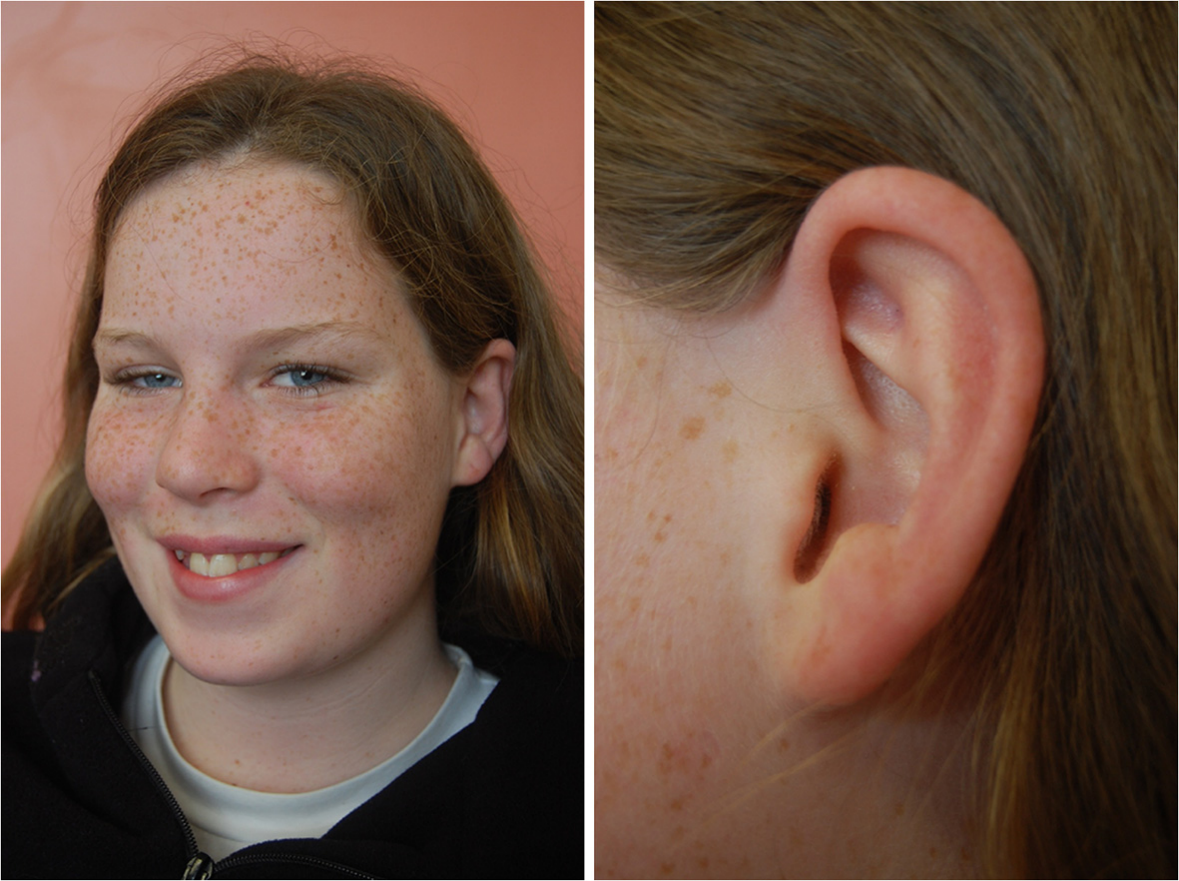
Clinical presentation of the subject at the age of 13.5 years.

The Caucasian female (46 XX) was conceived naturally and born by Caesarean section to a 19 year old, ex-smoking mother (BMI 21kg/m^2^), after a pregnancy complicated by urinary tract infection in the first month, and hyperemesis in the third trimester. There was no family history of BWS. Birth weight was 2.19 kg at 38 weeks gestation, 2.44 standard deviation score (SDS) below the mean in normal subjects, and placental weight 250g (1.7 SDS below the mean in ABC study), with normal placental examination and histology. She had no neonatal morbidity and no features of either SRS or BWS, other than a small umbilical hernia and transient neonatal hypoglycemia. Hypoglycemia (1.0mmol/L) was detected 2 hours after birth, which was treated with two infant formula feeds and blood glucose at 4 hours was 3.6mmol/L. Her postnatal intellectual development and growth was normal, achieving height between the 25^th^ and 50^th^ centile, weight between 50^th^ and 75^th^ centile at 13.5 years. Full examination by an experienced paediatrician (EM) was entirely normal at this age, in particular with no dysmorphic features, no hemihypertrophy, normal skin and ear creases (Figure 1). Chest X-ray, and renal ultrasound appearances were normal at the age of 13 years.

## Methods

We used DNA extracted from blood leucocytes using Qiagen’s DNA extraction kit. The methylation status at H19 and KvDMR was investigated by multiplex ligation dependent probe amplification (MLPA) using the SALSA MLPA ME030-B1 BWS/RSS kit (lot 0208) purchased from MRC-Holland (Amsterdam, The Netherlands) using the manufacturer’s instructions. In brief, approximately 100ng genomic DNA was first denatured and hybridised overnight with the probe mixture supplied with the kit, and then, after splitting the sample in two, treated either with ligase alone or with ligase and HhaI. PCR reactions were then performed with the reagents and primers supplied in the kit. The PCR products were separated on a 36cm capillary (model 3130XL, Applied Biosystems Foster City, CA, USA). The electropherograms were analyzed using Genescan software, and the relative peak area was calculated using the Coffalyser version 9.4 software (MRC-Holland). The single outlying result for methylation at ICR2, below three SD from the mean normalized ratios, was confirmed using methylation-specific PCR using bisulphite treated DNA as previously described [6], and further interrogated with MS-PCR for imprinting abnormalities at 8 other imprinted loci (PLAG1 (6q24), IGF2R (6q27), GRB10 (7p12), PEG1/MEST (7q32), DLK1 (14q32), SNRPN (15q11); PEG3 (19q32), NESPAS/GNAS (20q13)).

## Molecular genetic results

**Figure 2:**
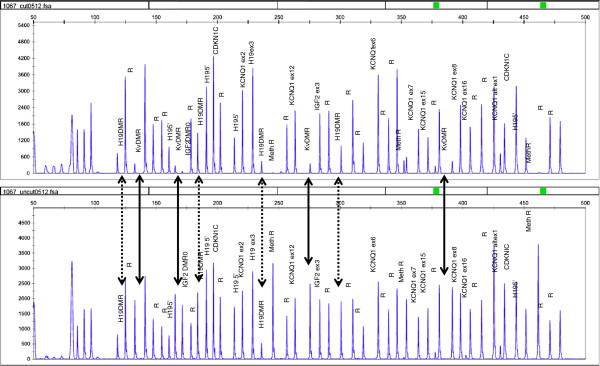
**Capillary electrophoresis pattern generated from the methylation specific MLPA reaction using the BWS/RSS probemix.** Upper panel indicates the result from the digestion reaction in the proband leucocyte sample, the lower panel indicates the result from the non-digested proband leucocyte sample. Dashed arrows indicate the four different CpG sites in the H19DMR and the solid arrows indicate the four different CpG sites in the KvDMR. Greater than 50% signal reduction at these sites indicate loss of methylation, which is observed at KvDMR but not at H19DMR.

**Figure 3:**
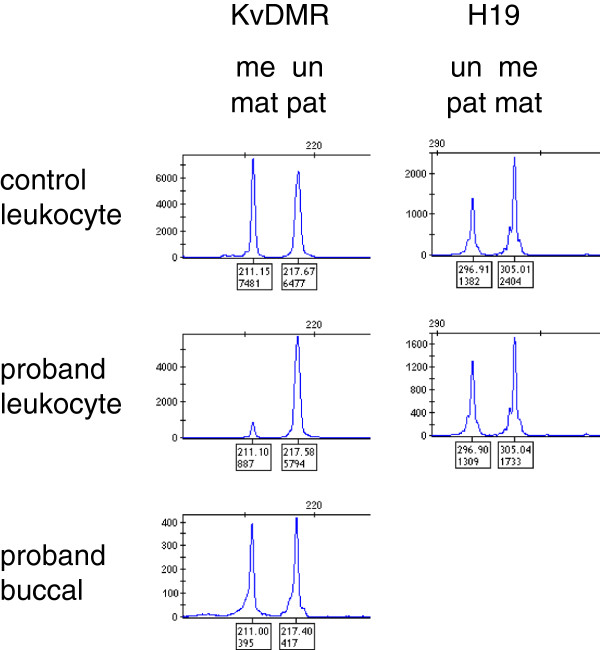
**Electropherograms of methylation-specific PCR amplicons for imprinting control regions 1 and 2 (ICR1 and ICR2) corresponding to H19 differentially methylated region (H19DMR).** The amplicon is identified above the first column as KvDMR and H19DMR above the second column, as are the methylated (me) and unmethylated (un) peaks corresponding to maternal (mat) or paternal (pat) origin respectively. Top row result from control leucocyte sample, middle row from proband leucocyte sample and bottom row result from proband buccal sample within the same experiment. Presence of both peaks at equivalent abundance to the normal control is consistent with a normal methylation profile; reduction of the maternal peak height at KvDMR indicates relative hypomethylation at this site as seen in the proband leucocyte DNA.

MLPA analysis of leucocyte derived DNA showed normal DNA methylation at ICR1 (H19 DMR) but marked LOM at ICR2 (KvDMR) (Figure 2); Individual methylation values for the four probes in KvDMR was 9% at 7171-L6780, 10% at 6276-L5782, 26% at 7172-L6781 and 11% at 7173-L6782. Individual methylation values for the four probes in H19DMR was 77% at 8743-L8763, 52% at 8744-L8764, 66% at 11080-L11762, 41% at 6266-L5772. LOM of ICR2 from leucocyte DNA but not buccal DNA was confirmed using MS-PCR (Figure 3). Testing for imprinting abnormalities at 8 other imprinted loci (PLAG1 (6q24), IGF2R (6q27), GRB10 (7p12), PEG1/MEST (7q32), DLK1 (14q32), SNRPN (15q11); PEG3 (19q32), NESPAS/GNAS (20q13) using MS-PCR was normal.

## Discussion

Our finding of LOM at ICR2 in leucocyte DNA but not in buccal DNA in a normally conceived, healthy child born small for gestational age is a novel observation and is important for two main reasons: Firstly, detecting a BWS epigenotype in blood but not buccal DNA in a child without clinical features of either BWS or SRS adds to the clinical spectrum of those found to have LOM ICR2. Secondly, given we screened 366 healthy, normally conceived children to find one case of LOM at ICR2, the prevalence of this epimutation is rare in the general population.

There are three possible explanations for our finding of isolated LOM at ICR2 in blood but not buccal DNA in an asymptomatic girl born SGA: (1) her result is part of the epigenetic variation within ICR2 seen in the normal population, including the 10% born SGA (2) she is sufficiently mosaic for the BWS epigenotype that it did not influence the tissues giving key presenting features (3) she is a surviving monozygotic (MZ) twin of a true BWS case.

Epigenetic variation at ICR2 in the general population has not been well described. Our molecular genetic result may simply represent a rare finding of LOM of ICR2 in blood but not buccal DNA in a normal 13 year old child who was born SGA. LOM of ICR2 has been found in blood DNA from 3/18 clinically normal children conceived by assisted reproductive therapies [11]. Two out of 79 cases with suspected SRS, were found to have LOM of ICR2, one of whom had additional LOM of ICR1 in peripheral blood [6]. Each of these cases may represent non-pathological variation at ICR2 observed in blood DNA.

BWS is a heterogeneous clinical disorder [12]. Our case born SGA had two minor clinical associations observed in BWS: a small umbilical hernia and transient neonatal hypoglycemia. These could represent an influence of ICR2 LOM, but it is also possible that both these relatively common conditions occurred by chance alone. The incidence of transient hypoglycemia in those born SGA is estimated at 25% [13] and umbilical hernia at 4.1% of Caucasian babies born at term [14]. Our case did not meet generally accepted criteria for clinical diagnosis of BWS based on 2–3 major findings (pre-/postnatal overgrowth, macroglossia, abdominal wall defects, anterior ear lobe creases and/or posterior helical pits, visceromegaly, childhood embryonal tumor, hemihyperplasia, renal abnormalities, positive family history of BWS, cleft palate) [12]. In contrast to the macrosomia at birth and rapid early childhood growth seen in BWS, our case was born SGA, and her height remained at the lower 25 percentile range until the age of 13 years. The prevalence of LOM at ICR2 in those with minimal clinical features of BWS such as umbilical hernia and transient neonatal hypoglycemia has not previously been reported.

The most likely explanation for the discordant epigenetic result observed in leucocyte derived DNA but not buccal DNA is that she is the surviving MZ twin of a true BWS case, which would also explain her low birthweight [15]. The incidence of monozygotic twinning in BWS is tenfold higher than population levels, and they are almost exclusively female [16]. Loss of one twin occurs in up to 30% of twin pregnancies conceived by assisted reproductive techniques [17,18], and may not be detected in most normally conceived cases due to lack of early ultrasound detection of twin pregnancies and complete reabsorption. Abnormal epigenetic results in leucocyte DNA, but not buccal DNA, have been described in the normal monozygotic twin of probands with BWS or SRS [4,19]. In this setting, fetal sharing of hematopoietic cells circulation in MZ monochorionic twins [16] or the migration of blood cell precursor cells from the common yolk sac [19] has been proposed.

Similar to our case who was healthy other than SGA, transient neonatal hypoglycemia and umbilical hernia, other cases of the healthy MZ twin of BWS cases have also shown some signs of BWS such as macrosomia, abdominal wall defects, and transient neonatal hypoglycemia [19-21]. The BWS features present in less affected twins may be explained by a low level of mosaicism in certain tissues of these individuals (in which case the number of aberrant cells in buccal swabs are simply too low to be detected) or that these features are caused by altered expression in the circulation of genes controlled by ICR2 (and that the only cells with the methylation defect in these individuals are in the circulation). The high rate of LOM of ICR2 observed in the blood may reflect the selective growth advantage for hematopoietic cells having this defect, whereas, studies in fibroblasts of discordant BWS twins has always only shown the epigenetic change only in the affected twin, favoring the latter explanation [19].

## Conclusion

Our naturally-conceived girl born small for gestational age adds to the clinical spectrum reported for LOM of ICR2 associated with BWS, observed in leucocyte but not buccal DNA.

## Consent

Written informed consent was obtained from the patient for publication of this Case report and the accompanying images. A copy of the written consent is available for review by the Series Editor of this journal.

## Abbreviations

BWS: Beckwith Wiedemann syndrome; DNA: Deoxyribonucleic acid; ICR: Imprinting control region; DMR: Differentially methylated region; LOM: Loss of methylation; MLPA: Multiplex ligation mediated probe amplification; MZ: Monozygotic; SDS: Standard deviation score; SRS: Silver Russell syndrome.

## Competing interests

The authors declare that they have no competing interests.

## Authors’ contributions

RM conceived of the study, carried out the analyses and drafted the manuscript. DM performed the molecular genetic analyses and contributed to the interpretation. EM assessed the patient and contributed to the interpretation. All authors read and approved the final manuscript.

## Pre-publication history

The pre-publication history for this paper can be accessed here:

http://www.biomedcentral.com/1471-2350/13/99/prepub
